# Antimicrobial resistance detection methods in water environments: a scoping review

**DOI:** 10.1093/sumbio/qvae034

**Published:** 2024-11-28

**Authors:** Zina Alfahl, Alexandra Chueiri, Shaunagh Carolan, Gabriel Darcy, Nadia Hussain, Niamh Cahill, Louise O’Connor

**Affiliations:** Antimicrobial Resistance and Microbial Ecology Group, School of Medicine, University of Galway, H91 TK33, Galway, Ireland; Centre for One Health, Ryan Institute, University of Galway, H91 TK33, Galway, Ireland; Centre for One Health, Ryan Institute, University of Galway, H91 TK33, Galway, Ireland; Molecular Diagnostics Research Group, College of Science & Engineering, University of Galway, H91 TK33, Galway, Ireland; Centre for One Health, Ryan Institute, University of Galway, H91 TK33, Galway, Ireland; Molecular Diagnostics Research Group, College of Science & Engineering, University of Galway, H91 TK33, Galway, Ireland; Centre for One Health, Ryan Institute, University of Galway, H91 TK33, Galway, Ireland; Molecular Diagnostics Research Group, College of Science & Engineering, University of Galway, H91 TK33, Galway, Ireland; Department of Pharmaceutical Sciences, College of Pharmacy, Al Ain University, PO Box 6414, Alain, UAE; AAU Health and Biomedical Research Center, Al Ain University, PO Box 64141, Abu Dhabi, UAE; Antimicrobial Resistance and Microbial Ecology Group, School of Medicine, University of Galway, H91 TK33, Galway, Ireland; Centre for One Health, Ryan Institute, University of Galway, H91 TK33, Galway, Ireland; Centre for One Health, Ryan Institute, University of Galway, H91 TK33, Galway, Ireland; Molecular Diagnostics Research Group, College of Science & Engineering, University of Galway, H91 TK33, Galway, Ireland

**Keywords:** antimicrobial resistance, traditional culture, water, molecular testing, sequencing

## Abstract

Antimicrobial resistance (AMR) in water environments poses a significant threat to public health, ecosystem stability, and the effectiveness of antimicrobial treatments. This review aims to provide a comprehensive overview of the methods used to detect AMR in various water environments. A literature search was conducted following the PRISMA guidelines. Original articles published in English relating to AMR in water environments were included. Reviews, protocols, and abstracts were excluded. A total of 115 publications were selected for full-text evaluation. Overall, river water samples were the most commonly assessed samples across all of the reviewed studies (49/115 studies, 42%). The top 3 countries investigating AMR genes in water samples were the USA (19 studies, 17%), China (11 studies, 10%), and Brazil (10 studies, 9%). The review revealed that polymerase chain reaction and metagenomic methods are increasingly preferred for their high sensitivity, specificity, and comprehensive detection capabilities, appearing in 65/115 (57%) and 31/115 (27%) studies, respectively. Despite higher costs and technical complexity, these methods provide valuable insights into the resistome of water environments. Culture-dependent methods, while most cost effective and straightforward, are limited by their time-consuming nature and inability to detect non-viable resistant organisms, reducing their effectiveness in comprehensive AMR surveillance. The review addresses the challenges and limitations of current detection methods and proposes directions for future research to develop more robust, cost-effective, and user-friendly detection methods. The review highlights the urgent need for integrated approaches to monitor and mitigate AMR in water environments, ensuring better public health and environmental protection.

**Sustainability Statement**

This review critically assesses current methodologies for detecting antimicrobial resistance (AMR) in water, crucial for addressing global health and environmental challenges. It supports UN Sustainable Development Goals 6 (clean water and sanitation) and SDG 3 (good health and well-being) by enhancing water quality management and mitigating health risks associated with AMR. By synthesizing existing knowledge, the review informs current challenges and future perspectives, promoting sustainable water management and public health outcomes. It underscores the importance of collaborative efforts SDG 17 (partnerships for the goals), in achieving these objectives. Ultimately, the review contributes to resilient infrastructure and sustainable development, ensuring a healthier environment and population globally.

## Introduction

Antimicrobial Resistance (AMR) is the capacity of pathogenic microorganisms to survive the inhibitory or lethal effects of antimicrobial agents (Reygaert 2018). The phenomenon of AMR has devastating effects, including a reduction in the therapeutic efficacy and potency of antimicrobials, leading it to be declared by the World Health Organization (WHO) as one of the biggest threats to public health. According to the WHO, AMR is considered as ‘the silent pandemic’, and is on the WHO list of the top 10 global public health threats facing humanity (WHO 2023). AMR has been driven by the excessive use of antimicrobials in human and veterinary medicine, agriculture, farming, and the subsequent contamination of the environment with antimicrobial drugs and resistant bacteria (Walsh et al. 2023).

AMR (both organisms and genes) can spread through humans, animals, and the environment (air and water), highlighting the importance of the One Health approach in addressing the interconnectedness of human, animal, and environmental health. Environmental spread of AMR is of particular concern in regions that have limited access to water, sanitation, and hygiene. While AMR monitoring has gained interest in clinical and veterinary settings, research on AMR monitoring in the environment remains limited (Velazquez-Meza et al. 2022). To understand the dynamics of AMR, it is vital to approach the tracking of resistant bacteria and resistance genes across relevant environments in a systematic approach. Examining the environmental aspect of AMR is pivotal to identifying and understanding the selection and dissemination pathways of resistant pathogens, particularly as they can significantly impact human health (Bengtsson-Palme et al. 2023).

In agriculture, routine antimicrobials are used to prevent and treat infectious disease outbreaks. The routine use of antimicrobials, mostly at sub-therapeutic doses, eventually leech into the feces of such animals (Martin et al. 2020), and subsequently can negatively impact plant biomes, soils, and waterways. In addition, other sources of antimicrobial contamination in our water environments, which pose a significant environmental threat, include waste from hospitals and pharmaceutical companies (Kayode-Afolayan et al. 2022). These sources require comprehensive monitoring to evaluate the impact of exposing humans to waterways where antimicrobials, multidrug-resistant (MDR) microorganisms, and antimicrobial resistance genes (ARGs) are present. The effectiveness of any corrective measures put in place would also require extensive monitoring to ensure reduction in human exposure to AMR from contaminated plants, animal products and specifically waterways (Bonetta et al. 2023). The main concern about the presence of antimicrobials in water bodies is their role in the persistence and dissemination of AMR in the environment, which facilitates the spread of resistance bacteria and genes from environmental microbes to human or animal pathogens. Conventional water treatments are only partially effective in removing antimicrobials and although the concentration levels of these chemicals in wastewater treatment plant effluents and surface waters are estimated to be low (ng/L to µg/L levels), they could still promote AMR development (Duarte et al. 2022). The significant role of water in the spread and persistence of AMR necessitates future studies to focus on this aspect.

Antimicrobial-resistant bacteria (ARB) constitute a significant category within AMR organisms, and unravelling the transmission dynamics of ARB, including the resistance determinants and mobile genetic elements they carry, presents a complex challenge. This intricacy is, in part, attributed to ARGs being carried on versatile mobile genetic elements with a broad host range, enabling them to transfer between different bacterial species. Furthermore, the ubiquitous nature and adaptability of bacteria contribute to their ability to survive in diverse environments, even when exposed to various stressors (Leonard et al. 2022). To date, several methods are available to identify resistant bacteria, which can be categorized as phenotypic resistance and genotypic resistance. Phenotypic resistance refers to the observable characteristics of bacteria that confer resistance, often assessed through methods like antibiograms that measure the effectiveness of antibiotics against cultured pathogens. This traditional approach involves culturing these pathogens under specified conditions, which, while straightforward, has limitations, such as the extended time required to replicate environmental conditions and the challenges associated with culturing viable microbes that do not grow easily (Muntean et al. 2022). On the other hand, genotypic resistance pertains to the identification of specific resistance genes, including potential gene regulatory mechanisms, which can be explored through molecular techniques such as PCR and sequencing. By combining both phenotypic and genotypic methods, a more comprehensive understanding of antimicrobial resistance can be achieved (Muntean et al. 2022). Advances in genotyping methods, such as polymerase chain reaction (PCR), random amplified polymorphic DNA (RAPD), PCR combined with restriction fragment length polymorphism (PCR–RFLP) and whole-genome sequencing (WGS), have shown great promise in the detection of ARGs (Galhano et al. 2021). The aim of this review is to consolidate methodologies employed in the identification of AMR, both organisms and genes, in natural water environments, encompassing both traditional and molecular approaches. Emphasis will be placed on delineating the strengths and weaknesses inherent in these methods and evaluating the efficacy and reliability of their outcomes.

## Methods

### Protocol

A protocol was developed prior to conducting this scoping review as per the JBI guidelines (Peters MDJ et al. 2020). Findings were reported according to the PRISMA (Preferred Reporting Items for Systematic Reviews and Meta-Analyses) guidelines (Moher et al. 2009).

### Data sources and searches

Three authors (A.C., S.C., and G.D.) independently searched the following databases: PubMed, ScienceDirect, Web of Science, and Google Scholar (for search strategy, see Appendix 1, Table S1). Reviews and references from included studies were manually searched to identify additional studies. The literature search included all studies published between 01 December 2013 and 31 December 2023.

### Study selection and data extraction

A.C., S.C., and G.D. independently screened articles by title and abstracts against the inclusion criteria and full text was subsequently reviewed. Inclusion criteria included original scientific articles that were published between December 2013 and December 2023, studies published in English language and nly water environment studies. Reviews, protocols, and abstracts were excluded. Abstracts were considered for inclusion if there was adequate information regarding study methods and results. Excluded papers were recorded with a rationale. A.C., S.C., and G.D. extracted the data, including the following: author, year of publication, study aim, sample type, number of samples, detection methods, and target genes. Z.A. was consulted to resolve any disagreements.

### Data extraction process and data items

Data extraction forms were developed from the template provided by the JBI guidelines (Peters 2020). Five articles were initially trialled with the data charting forms and results were reviewed by authors. Data was then extracted from the articles into a Microsoft Excel file and organized under the relevant headings by the lead author. Once the process was complete, the data was again reviewed and displayed in tabular form.

## Results

### Selection of sources of evidence

**Figure 1.**
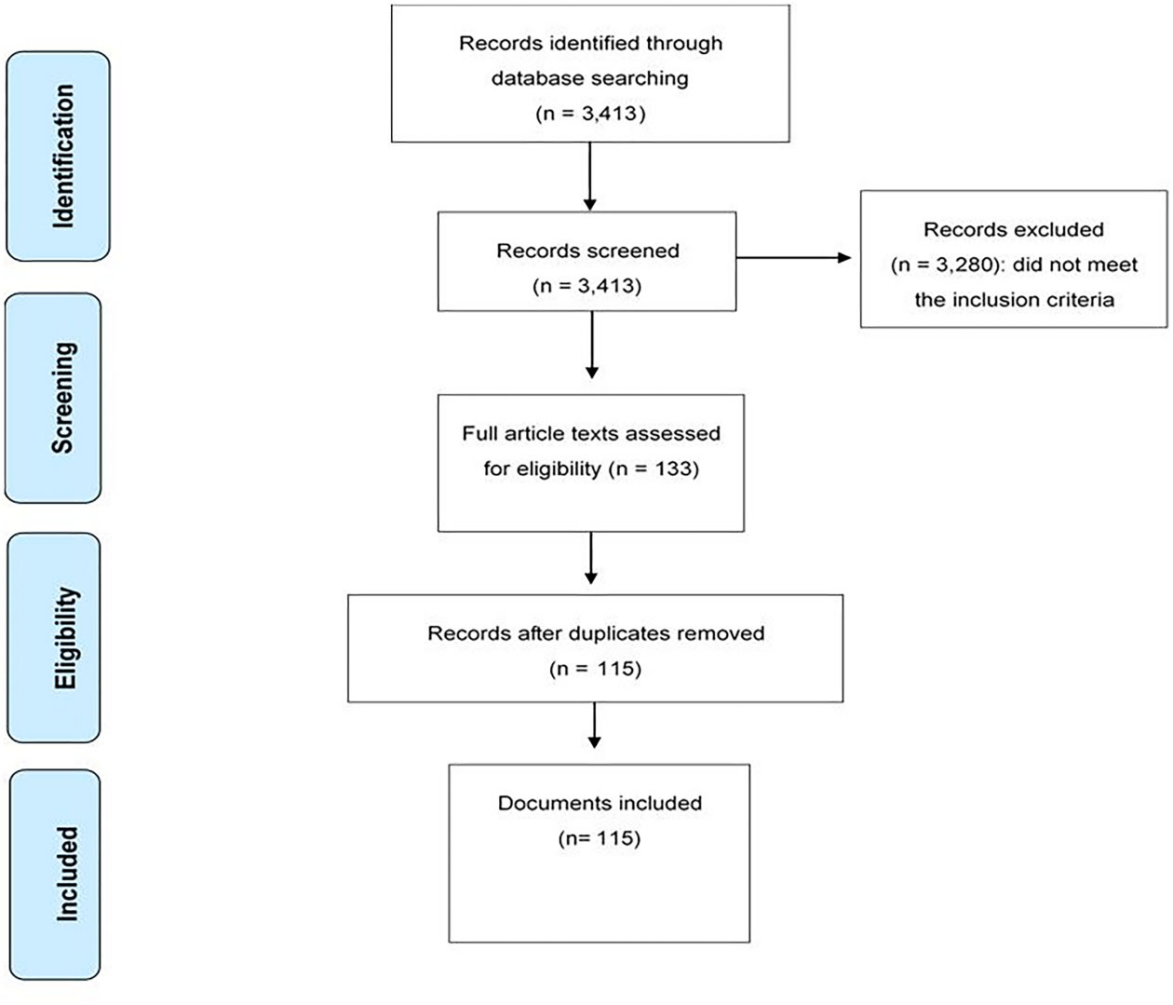
Flowchart of new studies (published between 01 December 2013 and 31 December 2023) included in the review based on the PRISMA protocol.

The database search produced 3413 articles, which were subsequently screened against the inclusion and exclusion criteria. The titles and abstracts of all studies were screened and a total of 3280 articles were excluded as they did not meet the inclusion criteria. Following a review and removal of duplicate articles, a total of 115 articles remained for inclusion in this review (Fig. 1).

### Characteristics of sources of evidence

Table 1 provides a summary of the studies included. Overall, river water samples were the most commonly tested samples with 49 out of the 115 studies reviewed (42%) assessing this sample type. This was followed by wastewater (32%) and groundwater samples (11%). Overall, culture-dependent methods were the most common method used in the detection of ARGs across the 115 studies reviewed, as 61% of studies employed this method. This was followed by PCR in 57% (*n *= 65) studies and sequencing techniques in 27% (*n* = 31) of the studies reviewed. The top 3 countries that tested water samples for the presence of ARGs were the USA (19/115 studies, 17%), China (11/115 studies, 10%), and Brazil (10/115 studies, 9%).

**Table 1.** General characteristics of selected studies.

Author, Year	Country	Study aims	Sample type	Number of samples	Detection methods			AMR related target genes
					Culture-dependent	PCR	Sequencing	
(Bessa et al. 2014)	Portugal	To evaluate the microbial quality of a polluted Portuguese river by measuring faecal contaminants and their antimicrobial resistance over a 3-month period.	Rivers	24	X	X		*vanA, vanB, vanC-1 vanC-2, vanC-3*
(Akiba et al. 2016)	India	To investigate the distribution of ESC- and carbapenem-resistant *Escherichia coli* in India’s aquatic environments and sewage treatment plants and compare plasmid DNA sequences to understand the nationwide spread of antimicrobial resistance determinants.	Rivers and Sewage Treatment Plants	74	X	X	X	*bla*_CTX-M_, *bla*_NDM_
(Kaiser et al. 2023)	USA	To define a clinical and urban resistome profile in an urban karst groundwater system in Bowling Green, Kentucky, using qPCR analysis of weekly samples from ten sites.	Urban Groundwater	443		X		Multiple target genes (see Kaiser et al. 2023, Supplement 1)
(Andrade et al. 2023)	Ireland	To investigate co-selective pressures in rural groundwater in Ireland and analysed groundwater-derived *E. coli* via whole genome sequencing to assess pathogenic potential.	Domestic Groundwater	250			X	*strA, aph(6), strA, bla_TEM_, sul2, tetA, floR, dfrA5, tetB, tetY*
(Nasri et al. 2017)	Tunisia	To quantify the abundance of genes encoding carbapenemase resistance (*blaKPC, blaNDM, blaOXA-48*-like) in wastewater effluents from Tunisian hospitals.	Wastewater	63		X		*bla*_KPC_, *bla*_NDM_ *bla*_OXA-48_
(Blaak et al. 2021)	The Netherlands	To analyse CPE prevalence in Dutch wastewater, investigate its association with WWTP characteristics, and assess WWTPs as potential sources of CPE based on treatment efficiency.	Municipal wastewater	200		X		*bla_CTX-M_ bla_TEM_ and/or bla_SHV_-genes, bla*_OXA-48_ *bla*_KPC_, *bla*_NDM_, *bla*_VIM_
(Kneis et al. 2019)	Germany	To use metagenomics to detect colistin resistance genes in raw municipal wastewater.	Municipal wastewater	14		X		*mcr-1, mcr-3, mcr-4, mcr-5, mcr-7, tetM*
(Ripanda et al. 2023)	Tanzania	To investigate the occurrence of antibiotic-resistant microbes in aquatic environments by isolating and assessing their antibiotic ARGs from selected isolates to inform public health measures.	Wastewater, river, and pond	57	X	X		*bla_SHV_, bla_CTX-M_ tetA*, *tetB, tetD, sul1, sul2*
(Li et al. 2022)	China	To understand AMR extent and genetics in hospital wastewater pathogens, evaluate treatment’s AMR removal, assess ‘last-resort’ antibiotic-resistant pathogen prevalence, and evaluate nosocomial MDR infection potential.	Hospital wastewater	8	X	X		Multiple target genes^1^
(Nolan et al. 2023b)	Ireland	To investigate whether faecal pollution increased ARG levels in phages along three rivers in Dublin, finding that such pollution indeed raised phage-associated ARGs, emphasizing the role of phages as reservoirs of resistance genes in aquatic environments.	Rivers	231		X		*bla_TEM_, qnrS, tetO, sul1*
(Goh et al. 2022)	Singapore	To investigate ARBs and ARGs in natural aquatic environments and develop a modeling framework to assess the relative AMR burden, ultimately identifying AMR hotspots for improved control and management.	Lakes and tributaries	40		X		*bla_KPC_, bla_NDM._ bla_SHV_ bla_CTX-M_, vanA, tetO* *tetM, qnrA*
(Bell et al. 2023)	Bangladesh	To examine seasonal, crop type (fish), and geographical differences in microbial assemblages, ARG abundance, and diversity in Bangladeshi aquaculture pond water using metagenomic shotgun DNA sequencing.	Aquaculture ponds	24			X	Multiple target genes (See Bell et al. 2023, supplementary data)
(Rubin-Blum et al. 2023)	Israel	To understand AMR diversity, sources, transmission mechanisms, and seasonal dynamics, we collected Alexandr River water samples across different seasons, sequenced environmental DNA, and analysed metagenomes to link AMR mechanisms to taxonomy and identify ARG transmission potential.	River	18			X	Multiple target genes (see Rubin-Blum et al. 2023, supplementary data)
(Chaturvedi et al. 2021)	India	To investigate the spread of ARGs, efflux genes, and HMRGs in anthropogenically impacted Indian rivers, focusing on MDR, β -lactams, mobile genetic elements, and efflux genes across various geo-climatic zones.	Rivers	4	X	X		*bla*_CTX-M_,*bla*_TEM_, *bla*_SHV_, *bla*_OXA-48_
(Rothman et al. 2023)	USA	To investigate transcriptomic diversity, active AMR gene transcription, conserved biochemical pathways, and large-scale	Municipal wastewater	275			X	Multiple target genes (see Rothman et al. 2023, Fig. 3)
		temporal patterns of microbial transcription in Southern California wastewater.						
(Asaduzzaman et al. 2022)	Bangladesh	To quantify and visualize the prevalence and abundance of AMR organisms and ARGs in urban and rural Bangladesh using an integrated spatial approach and GIS.	Wastewater, rivers, ponds, and drinking water	397	X	X		*bla*_NDM-1_, *bla*_CTX-M-1_
(Conte et al. 2017)	Brazil	To evaluate β-lactam and quinolone resistance in *E. coli* and *Klebsiella pneumon iae* and assess ciprofloxacin residue in hospital sewage, WWTP, and river water samples.	Wastewater	7	X	X		Multiple target genes^2^
(Nakayama et al. 2017)	Vietnam	To elucidate antimicrobial residues, ARGs, and water microbiota at Mekong Delta aquaculture sites to aid Vietnamese water quality policy development.	Aquacultures and rivers	12		X		*bla_CTX-M-1_, bla_CTX-M-9_, bla_TEM_, bla_SHV_, tetC, sul1, sul2*
(Shahin et al. 2019)	Iran	To investigate on Shigella spp. isolated from water samples, including antimicrobial resistance phenotypes, associated ARGs, and integrons, despite extensive research on Shigella in clinical samples and food products worldwide.	Surface, ground, and farm waters	788	X	X		Multiple target genes^3^
(Sevillano et al. 2020)	UK and Netherlands	To survey ARGs in DWDS, assess their abundance in relation to disinfectant residual, determine host-association of high prevalence ARGs, and evaluate their relationship with disinfectant presence/concentration in DWDS.	Drinking water systems	39			X	Multiple target genes (see Sevillano et al. 2020, supplementary data)
(Zagui et al. 2023)	Brazil	To assess the occurrence of MDR Enterobacter spp. in aquatic matrices and investigate their genotype for β-lactam antibiotic resistance and metal tolerance due to their pathogenic potential for humans and animals.	Wastewater and surface water		X	X		Multiple target genes^4^
(Jeamsripong et al. 2022)	Thailand	To analyse AMR, ESBL production, and virulence genes in *E. coli*, Salmonella spp., *V. cholerae*, and *V. parahaemolyticus* from coastal water, and assess their association in coastal aquaculture regions.	Aquaculture		X	X		Multiple target genes^5^
(Tajbakhsh et al. 2015)	Iran	To investigate the prevalence of class 1 and 2 integrons in multi-drug resistant *E. coli* isolated from aquaculture water.	Aquaculture	150	X	X		*cmlA, aac(3), IIa, tetA, qnrA, Sul1, Sul2*
(Sabri et al. 2020)	Netherlands	To examine emerging contaminants and nutrients along a Dutch river, studying the impact of WWTP effluent by sampling from 0.5 km upstream to 20 km downstream over a year to correlate ARGs with environmental factors.	River	350		X		*ermB, sul1, sul2, tetW*
(Su et al. 2018)	China	To investigate ARG abundance in source and tap water, their relationship with bacterial communities, and their removal in DWTPs, offering insights into ARG diversity, dissemination, and removal in drinking water systems.	Source and Tap Water	51		X	X	Multiple target genes^6^
(Rodriguez-Mozaz et al. 2015)	Spain	To assess the pollution level of antibiotics and ARGs from hospital and urban wastewaters, their removal by WWTPs, and their presence in the receiving river.	River	15		X		*bla_TEM_, ermB, qnrS, sul1, tetW*
(Wang et al. 2020)	China	To understand the spatiotemporal variation of ARGs, assess the impact of water quality and microbial communities on their distribution, and provide insights for developing strategies to control ARG pollution in agriculturally disturbed lakes.	Lakes, rivers, and aquaculture ponds	16		X	X	*tetA, tetB, tetC, sul1, sul2, qnrs*
(Cacace et al. 2019)	France, Italy, Norway, Portugal, Germany, Netherland, Cyprus, Turkey, Austria, and UK	To quantify selected ARGs and i*ntI1* in WWTP effluents and receiving waters across Europe and assess the potential impact of WWTPs on these water bodies.	Wastewaters and receiving waters	120		X		*bla_TEM_, bla_OXA-48_, bla_OXA-58_, bla_CTX-M-15_, bla_CTX-M-32_, bla_KPC-3_, sul1, tetM, mcr-1*
(Bergeron et al. 2015)	USA	To examine the presence of antibiotic resistance genes in source, finished, and tap water, revealing numerous antibiotic-resistant bacteria and few antibiotic resistance genes in the source water.	Raw source and treated drinking water	108	X	X		*erm(B), sul1, tetA, tetW, tetX, mecA*
(Li et al. 2014)	USA	To investigate *S. enterica* prevalence in irrigation pond water on southeastern US produce farms, develop an improved isolation method, and compare it with the traditional method in the US FDA’s Bacteriological Analytical Manual.	Irrigation pond water	170	X			N/A
(Titilawo et al. 2015)	Nigeria	To assess the frequency of multi-drug resistant *E. coli* contamination in selected surface waters from Osun State, South-western Nigeria, to determine high-risk contamination.	Rivers	120	X			N/A
(Haberecht et al. 2019)	USA	To analyse environmental water samples for AMR *E. coli*, characterize their phenotypic and genotypic resistance profiles, and investigate the clonal relatedness of AMR *E. coli* strains across water systems.	Sewer water, wastewater treatment plant (WWTP) Influent, WWTP effluent and surface water	17	X	X	X	*bla*_OXA_, *bla*_CTX-M_, *bla*_TEM_
(Gómez et al. 2020)	Spain	To identify staphylococcal species in surface waters of La Rioja (northern Spain) and characterize their molecular profiles and antimicrobial resistance.	Surface water	62	X	X		Multiple target genes^7^
(Lyimo et al. 2016)	Tanzania	To assess the prevalence of antibiotic-resistant *E. coli* in surface waters in northern Tanzania.	Rivers, lakes, streams, ponds, taps, and bore-wells	155	X	X		*bla*_TEM-1_, *bla*_SHV-1_, *bla*_CTX-M_, tetA, tetB
(Blaak et al. 2015)	Netherlands	To analyse AMR *E. coli* prevalence and concentrations in Dutch surface water, integrating data from multiple studies, and explore wastewater’s role as a contamination source.	Rivers, canals, rivulets, lakes, the North Sea, and Wastewater	146	X	X		CTX-M_-group 1_, CTX-M_-group 2_, CTX-M_-group 9_ *bla*_OXA_, *bla*_SHV_, *bla*_TEM_
(Kaiser et al. 2022)	USA	To analyse ARB proliferation in urban-influenced karst groundwater systems of Bowling Green, Kentucky, and the Tampa Bay Metropolitan Area, Florida.	Groundwater	488	X	X		Multiple target genes^8^
(Moretto et al. 2021)	Brazil	To evaluate AMR enterobacteria and faecal contamination was assessed in four surface waters.	River	48	X	X		*bla_CTX-M_, bla_SHV_, bla_TEM_, bla_KPC_, bla_VIM_, bla_NDM_, bla_SPM_, bla_OXA-48_*
(Gabashvili et al. 2022)	Georgia	To conduct a pilot metagenomics study to explore the spectrum of antibiotic-resistant genes (ARGs) potentially present in the coastal recreational waters of the Black Sea around the City of Batumi.	Seawater	4			X	Multiple target genes (see Gabashvilli et al. 2022, supplementary data)
(Nguyen et al. 2020)	Vietnam	To understand the influence of natural bacterial hosts on the evolution, spread, and positive selection of acquired ARGs.	Canal water	10			X	Not specified
(Kimera et al. 2021)	Tanzania	To identify occurrences of ESBL-producing Enterobacteriaceae, carbapenem-resistant Enterobacteriaceae (CRE), and quinolone-resistant *E. coli* and Klebsiella spp.	River	196	X			N/A
(Elder et al. 2021)	UK	To undertake ARG speciation in a large river catchment in the South West of England.	River catchment	63		X		*qnrS, ermB, sul1, catA*
(Byrne et al. 2022)	Malawi	To develop a low-cost extraction method independent to commercial kits or reagents.	River	98	X	X		*blaCTXM-, blaSHV*
(Sulca et al. 2018)	Peru	To assess the prevalence of antimicrobial resistance in *Vibrio* spp. strains isolated from various monitoring points in Lima sea and investigate whether this resistance is linked to the presence of resistance integrons.	Sea water	46	X	X		*intI1, intI2, intI3, intI4, mrhB, intI4, MutT*
(Johnson et al. 2020)	India	To characterize the presence of *E. coli* and ESBL-producing *E. coli* in environmental water throughout Kanpur, India.	Open drains, canals, and rivers	32	X			N/A
(Yang et al. 2023)	China	To develop a nanopore-based metagenomic sequencing workflow for rapid and precise estimation of viable microbial cell counts in beach waters using propidium monoazide (PMA) and cellular spike-ins.	Beach water	4			X	Multiple target genes (see Yang et al. 2023, Table S16)
(Angeles et al. 2020)	Bangladesh	To investigate the presence of different chemical in water samples from urban and rural sites in Bangladesh.	Surface waters	30	X			N/A
(Castrignanò et al. 2020)	UK	To verify the occurrence and transformation of FQs, qnrS gene, and estimate public exposure using wastewater-based epidemiology.	Waste water and river water	91		X		*qnrS*
(Janssen et al. 2021)	Brazil	To describe a MDR NTCKPC-bearing *K. pneumoniae* strain belonging to a novel ST that has been found during a prospection for xenobiotic-degrading bacteria in an artificial urban lake, next to a WWTP.	Lake	1	X	X	X	*blaCTX-M-15, KPC-2, NTE_KPC_*
(Boggs et al. 2023)	USA	To develop culture-based methods to efficiently assess the presence/absence or density of tetracycline-resistant culturable *E. coli* with enhanced efficiency and reduced costs compared to current multi-step approaches.	Surface waters	61	X			N/A
(Ahsan et al. 2022)	Pakistan	To assess ESBL *E. coli* occurrence in Islamabad Capital Territory's wastewater and determine the percentage of *blaCTX-M, blaSHV*, and *blaTEM* genes to estimate antimicrobial resistance in *E. coli* isolates.	River and wastewater treatment plant	32	X	X		*blaTEM, blaSHV, blaCTX-M*
(Mpondo et al. 2021)	South Africa	To evaluate the occurrence and antibiogram profiles of Listeria species isolated from river water and irrigation sources in the Sarah Baartman District Municipality, South Africa.	River	6	X	X		
(Dungan and Bjorneberg 2021)	USA	To increase knowledge of the abundance and AMR of *E. coli* and enterococci in irrigation return flows (IRFs) of the Upper Snake River watershed in south-central Idaho.	River	174	X			
(Mahmud et al. 2020)	Bangladesh	To determine the prevalence of ESBL-producing, MDR, and virulent *E.coli* in drinking water samples.	Drinking water	421	X	X		Multiple target genes^9^
(Salazar et al. 2022)	Uruguay	To investigate if anthropogenic impact in urban environments increases antimicrobial-resistant genes and if sewage infrastructure affects MDR plasmid contamination.	Beaches, creeks, spillways and sewage collector pipes	44			X	Multiple target genes (see Salazar et al. 2022, Table S3)
(Lepuschitz et al. 2019)	Austria	To assess the diversity of ESBL and carbapenemase-producing *K. pneumoniae* in water samples from four major Austrian rivers and compare them with clinical isolates, identifying potential sources of anthropogenic pollution.	Rivers	10			X	Multiple target genes (see Lepuschitz et al. 2019, Table 2)
(George et al. 2022)	India	To obtain lytic coliphages from various water samples, which can serve as a promising alternative for controlling antibiotic-resistant *E. coli* and enhance the phage repository for these AMR pathogens.	Rivers, aquaculture ponds, lake, sewage treatment plant, domestic waste and canals	30			X	Multiple target genes (see George et al. 2022, supplementary data)
(Mahmoud et al. 2020)	Sudan	To determine the prevalence of carbapenem-resistant genes among *E. coli* isolated from drinking water.	Taps, cooler, and tanks in houses	150	X	X		*bla_NDM_, bla_IMP_, bla_VIM_, bla_SPM_, bla_KPC_, bla_OXA-48_*
(Nowicki et al. 2021)	Kenya	To understand non-clinical *E. coli* populations in Kenya and provide insight into their occurrence in rural water systems. Compare point-of-collection (PoC) and point-of-use (PoU) water sources to assess the utility of *E. coli* as an indicator of fecal contamination in these settings.	Reservoir, borehole, and dug well	44			X	Multiple target genes^10^
(Neher et al. 2020)	USA	To compare AMR indicators in runoff, we examined two adjacent catchments, one manured and one non-manured. To compare three catchments (600–804 ha): one with manure influence, one control with no manure, and one with urban influences.	Watershed	235		X		Multiple target genes^11^
(Song et al. 2017)	UK	To show that RamanDIP and RACE are effective culture-independent methods for rapidly identifying antibiotic-resistant bacteria at the single-cell level in their natural environments.	River	2	X			N/A
(Tabut et al. 2022)	Thailand	To describe the antibiotic resistance of *E. coli* from surface water, wastewater, and discharge water in the Namsuay watershed in upper north east Thailand.	Surface water, wastewater, and discharge water	44	X	X		Multiple target genes^12^
(Singh et al. 2020)	India	To detect carbapenemase, ESBL and *intI1* gene in fermenting and non fermenting bacterial isolates from 128 water samples.	Sewages, rain water, ponds, canals, rivers, and tap sources	128	X	X		*bla_TEM_, bla_SHV_, bla_KPC_, bla_NDM_*
(Silva et al. 2021)	Portugal	To isolate Staphylococci from surface waters and to investigate the presence of ARGs and virulence factors as well as the genetic lineages of all *S. aureus* isolates.	Surface waters (rivers, streams, irrigation ditches, dams, lakes, and fountains)	78	X	X		Multiple target genes^13^
(Bartley et al. 2019)	Brazil	To conduct a point prevalence study to investigate whether surface water sampled from a typical urban setting in North east Brazil is a potential source of AMR.	River	32	X	X		Multiple target genes^14^
(Ahmed et al. 2022)	Ghana	To assess the presence of bacteria and antibiotic resistance among *E. coli* and *P. aeruginosa* isolates from different water sources from the Greater Accra Region of Ghana.	Sachets, bottled water, tap water, borehole, and well water	524	X			N/A
(Bhandari et al. 2023)	Australia	To characterize recent surveillance of environmental NOVC strains isolated from Queensland River waterways to understand their virulence, antimicrobial resistance profile and to place genetic current *V. cholera* strains from Australia in context with international strains.	River	10 sites			X	Not specified
(Williamson et al. 2022)	USA	To sequence 38 genomes collected from soil and water isolates in Flagstaff and Slovenia in an effort targeted towards environmental surveillance of *C. difficile*.	Puddle	50	X		X	Not specified
(Liao et al. 2021)	China	To evaluate the distribution of AMR genes and analyze the drug resistance of *E. coli* in aquaculture farms.	Surface water aquaculture farms	30	X	X		*bla_TEM_, bla_CIT_, bla_NDM_, floR, OptrA, cmlA, aphA1, Sul2, oqxA, and qnrS, bla_KPC_, bla_IMP_, cfr*
(Pek et al. 2023)	Singapore	To understand the AMR burden in recreational beach waters using extended-spectrum betalactamase *E. coli* (ESBL-EC) as an indicator.	Recreational beach waters	90	X			
(Tucker et al. 2022)	South Africa	To identify urban sectors contributing to AMR and potential hotspots by determining resistance profiles from agriculture, industry, hospitals, and	Urban water	10 sites	X		X	Multiple target genes (see Tucker et al. 2022, supplementary data)
		residential areas discharging sewage and greywater.						
(Takeda-Nishikawa et al. 2023)	Japan	To determine the prevalence of AMR in environmental surface water in Japan, we used DNA sequencing techniques on environmental water DNA (eDNA) and the DNA of multidrug-resistant bacteria (mrDNA)	River/pond water (below surface)	49 sites	X		X	Not specified
(Nolan et al. 2023a)	Ireland	To investigate the impact of different land uses on river in ecosystems.	River	212		X		*bla_TEM_, qnrS, tetO, sul1*
(Holton et al. 2022)	UK	To understand the spatiotemporal dynamics of antimicrobials in the River Eerste, upstream and downstream of the most impacted section, over 11 months. To identify the most persistent and prevalent antimicrobial groups, verify their fate and metabolites, and pinpoint contamination hotspots.	River	812		X		Not specified
(Afema et al. 2016)	USA and Uganda	To investigate potential sources of NTS in Kampala, Uganda by determining occurrence in human, livestock and environmental sources.	Channel drains	Not specified	X			
(Al-Sarawi et al. 2023)	Kuwait	To identify ARGs prevalence in *E. coli* isolated from mollusks and coastal water samples collected in a previous study.	Marine water	6		X	X	*intl1*
(Hornsby et al. 2022)	USA	To validate IDEXX substrate assay for detection and enumeration of cefotaxime resistant *E. coli* in multiple environmental reservoirs.	Surface water	27	X			N/A
(Liu et al. 2023)	China	To analyse the fugitive characteristics of 10 antibiotics of sulfonamides (sulfadiazine, sulfamethazine, sulfadimidine, sulfathiazole, sulfapyridine, sulfamethoxazole) and tetracyclines (tetracycline, oxytetracycline, chlortetracycline and doxycycline) in the coastal waters and surface sediments of the Yangtze River Estuary.	Coastal region along river (water and sediment)	32			X	Not specified
(Li et al. 2020)	China	To investigate the effects of eutrophication on AMR of water-borne pathogens	Surface water	40	X			
(Hu et al. 2023)	China	To elaborate the interaction between disinfection and environmental AMR, providing instructions for the proper use of disinfectants.	Surface water from lakes	20			X	Multiple target genes (see Hu et al. 2023, Fig. 2)
(Nolan et al. 2023b)	Ireland	To assess whether faecal pollution of three rivers was responsible for increased levels of ARGs in phage particles using established phage-faecal markers, focusing on four ARGs.	Rivers	231		X		*bla_TEM_, tetO, qnrS, sul1*
(Mughini-Gras et al. 2021)	Netherlands	To determine microplastic concentrations, their polymer types and associated microbial communities, including selected virulence and AMR genes, in the river Rhine.	River	8		X		*sul1, erm(B)*
(Enayati et al. 2015)	Iran	To determine the incidence of *Enterococcus* species and six virulence factors of *En. faecium* which were isolated from surface water and wells.	Wells and rivers	15	X			N/A
(Liguori et al. 2023)	USA	To compare trends and conclusions from enumerating cefotaxime-resistant *E. coli* versus quantifying *sul1* or *intI1* via qPCR across various water samples.	Wastewater, surface water and recycled water	14 wastewater (others not mentioned)	X	X		*sul1, intI1*
(Ramalho et al. 2022)	Brazil	To evaluate the occurrence and diversity of ARG in water sources from an urban centre, in Southern Brazil.	Pre/post drinking water treatment plant and sewage	32		X		*bla_TEM_, bla_SHV_, bla_CTX-M_ bla_KPC_, bla_NDM_, bla_OXA-48_, bla_GES_, bla_VIM_, bla_IMP_, mcr-1, qnrA, qnr, qnrS, sul1, sul2, tetA, tetB*
(Rebello and Regua-Mangia 2014)	Brazil	To test for *E. coli* contamination in aquatic ecosystems.	Residential, industrial, agricultural, hospital wastewaters, and recreational waters	Not specified	X			
(Ramírez Castillo et al. 2013)	Mexico	To test untreated and treated wastewater discharge into rivers and streams which may lead to waterborne infection outbreaks and spread antibiotic resistance genes significantly.	River and main tributaries	30	X	X		*gyrA, parC, qnrA, qnrB, qnrS, acc-(6′)-lb*
(Wei et al. 2017)	China	To determine prevalence and genetic diversity of *En. faecalis* isolates from mineral water and spring water in China.	Mineral and spring water factories	314	X			N/A
(Tsvetanova et al. 2022)	Bulgaria	To assess the prevalence of AMR among the heterotrophic bacteria in drinking water provided by a Bulgarian drinking water supply system.	Dam, treated water, public building, fountain, and residential homes	40	X	X		*tetA, _blaTEM-1_, sul1, cat, qrnS, dfrA7, intl*
(Gu et al. 2023)	China	To determine the occurrence and abundance of ARGs in a drinking water treatment plant was comprehensively investigated using metagenomics.	Drinking water treatment plant and river	6	X		X	Multiple target genes (see Gu et al. 2023, supplementary data 2.1)
(Hassan et al. 2023)	UK	To use comparative genomics to identify the prevalence of AMR genes.	Tap and pond	Not specified		X		*mcr-1, bla_KPC_, bla_VIM_, bla_OXA-48_, bla_OXA-23_*
(LaMontagne et al. 2023)	USA	To compare virulence gene prevalence between isolates resistant and susceptible to antibiotics	River	Not specified	X			N/A
(Palhares et al. 2014)	Brazil	To examine the *Salmonella serovars* and antimicrobial resistance within an animal-based agriculture river system.	Rivers	384	X			N/A
(Spänig et al. 2021)	Germany and China	To provide an estimation of the characteristic resistance of AMR in European lakes, which can be used as a comprehensive resistome dataset to facilitate and monitor temporal changes in the development of AMR in European freshwater lakes.	Lakes	274			X	Not specified
(Almakki et al. 2017)	France and Iraq	To develop an original method for characterizing the antimicrobial resistance of bacterial communities in coastal waters.	Near coastal river waters, lagoon brackish waters, and lake freshwaters	3	X			N/A
(Mala et al. 2017)	Thailand	To investigate the phylogenetic relationships of intSXT gene sequences from 31 clinical and 14 environmental *V ibrio cholerae* O1 and non-O1/non-O139 isolates and 11 sequences amplified directly from environmental water samples.	2 ponds, 7 canals, and 2 wastewater reservoirs	80		X		*intI1, int_SXT_, tetA, sul2, dfrA1, dfr18, strB, floR*
(Cho et al. 2020a)	USA	To determine mechanisms of antimicrobial resistance as well as the abundance and diversity of plasmids among MDR enterococci from surface water.	Surface waters	Not specified		X		Multiple target genes^15^
(Tsvetanova and Najdenski 2023)	Bulgaria	To quantify heterotrophic bacteria resistant to five antibiotic groups in the Yantra River and its tributary, assess AMR prevalence in Enterobacteriaceae, and to evaluate the impact of urban effluents and rural runoff on AMR at eight sampling points	Rivers	48	X			N/A
(Luczkiewicz et al. 2015)	Poland	To determine species distribution and antimicrobial susceptibility of cultivated *Pseudomonas* spp.	Bay	Not specified	X			N/A
(Kadykalo et al. 2020)	Canada	To explore antimicrobial resistance patterns in water samples collected from the Grand River watershed (southwestern Ontario, Canada) to describe the composition, trends and potential risks of AMR in the aquatic environment.	River	967	X			N/A
(Shaw et al. 2014)	USA	To evaluate antimicrobial evaluate antimicrobial *Vibrio vulnificus* and *V. parahaemolyticus* in the estuarine-marine environment.	Bays	9	X			N/A
(Snyman et al. 2021)	South Africa	To characterize mobile colistin resistance genes in river and storm water in regions of the Western Cape of South Africa.	Rivers and storm water	Not specified	X	X	X	*mcr-1/2/3/4/5/6/7/8/9*
(Igbinosa 2016)	Nigeria	To evaluate the presence of *Vibrio* isolates recovered from four different fish pond facilities in Nigeria and to determine their antibiogram profiles, and evaluate the public health implica- tions of these findings.	Ponds	56	X			
(Suzuki and Ushijima 2016)	Japan	To understand the distribution of antimicrobial resistant Salmonella in an urban river that flows through the provincial city of Miyazaki, Japan.	River	9	X			N/A
(Cho et al. 2020b)	USA	To investigate the role of surface water as a reservoir for antimicrobial-resistant enterococci by collecting and analysing water samples from the Upper Oconee watershed, Athens, over a 2-year period.	Rivers and streams	458	X			N/A
(Canellas et al. 2021)	Brazil	To evaluate the presence of potentially pathogenic and antimicrobial-resistant bacteria in a heavily polluted recreational estuarine ecosystem.	Tropical estuary	6	X	X		*bla*_CTX‐M‐8_, *bla*_CTX‐M‐1,2_, *bla*_CTX‐M‐14_, *bla*_GES_, *bla*_TEM_, *bla*_SHV_
(Czekalski et al. 2015)	Switzerland	To investigate the human impact on the presence and distribution of ARGs in aquatic environments.	Lakes	21		X		*sul1, sul2, tetB, tetM, tetW, qnrA*
(Solaiman et al. 2022)	USA	To investigate the impact of human activities on the presence of ARGs in aquatic environments.	River, creek, and pond	333	X			
(Aujoulat et al. 2021)	France	To explore antimicrobial resistance, particularly those of clinical concern, in urban rivers flowing in this urban area.	River	Not specified	X	X		*bla_SHV_, bla_TEM_, bla_CTX-M_*
(Alipour et al. 2014)	Iran	To examine the presence and the antibiotic resistance patterns of Enterococcus spp. isolated from the Babolrud River in Babol and coastal waters in Babolsar.	River and coastal waters	Not specified	X			N/A
(Drigo et al. 2021)	Australia, Greece	To better understand the efficacy of recycled water treatment plants (RWTPs) for removal of human opportunistic pathogens and antimicrobial resistant microorganisms.	Recycled water system	162		X		*qnrS, bla_SHV_, bla_TEM_, bla_GES_, bla_KPC_, bla_IMI_, bla_SME_, bla_NDM_, bla_VIM_, bla_IMP_, bla_OXA-48-_*_like_, *mcr-1,mcr-3, intl1*
(Adesiyan et al. 2019)	Nigeria, South Africa	To evaluate the prevalence of *Plesiomonas shigelloides* and their antibiogram fingerprints in water sample collected from four rivers in South-western Nigeria.	River	144	X	X	X	*sul1, sulII, dfr1, dfr, ampC, tetA, tetE, catII, cmlA1, aphA1, strB*
(Mukherjee et al. 2021)	USA	To test wastewater treatment plants for the dissemination of AMR and MDR.	Creek	36	X	X		*intI1, tetA, tetW, ampC, blaTEM, mecA, aacA*, and *ermA*
(Santos et al. 2023)	Brazil	To evaluate the occurrence, virulence markers and AMR in 468 drinking water samples from 15 public fountains located in four urban parks of São Paulo city.	Water fountains	468	X	X		*mecA*
(Cohen et al. 2020)	Israel	To assess the occurrence of CPE in nearshore aquatic bodies.	River and beach	8	X		X	Not specified

1 Genes detected blaTEM, blaCTX-M, blaROB-1,blaKPC, blaNDM-1, blaIMP-1, aph(3′’)-Ib, aadA1, aadA2, tetR, tet(X4), tetA, qnrA, qnrD, qnrS, oqxA, sul1, dfrA14, catI, floR, fosA3, mcr-1, mcr-2, vanA, vanB, vanM, ermB, mphE, arr-3, cfr(B), optrA, intI1, intI2, intI3.

2 Genes detected blaTEM, blaSHV, blaCTX-M, blaGES, blaPER, blaKPC, blaNDM, blaVIM, blaSIM, blaGIM, blaBES, blaVEB, blaSPM, blaIMP, blaOXA.

3 Genes detected blaTEM, blaSHV, blaCTX-M, blaOXA, blaPER, blaVEB, blaGES and blaCMY, blaKPC, blaNDM, blaIMP, qnrA, qnrB, qnrS, aac(60)-Ib, tet(A), tet(B), tet(C), tet(D).

4 Genes detected blaCTX-M group-1, 2, 8, 9, 25, blaPER, blaVEB, blaBEL, blaGES, blaKPC, blaIMP, blaNDM, blaVIM, blaOXA-48.

5 Genes detected blaTEM, blaSHV, blaCTX-M, blaPSE, blaNDM, blaOXA, floR, cmlA, ermB, qnrS, aadA1, tetA, tetB, strA, sul1, sul2, dfrA1, dfrA12, mcr-1, mcr-2, mcr-3.

6 Genes detected: sul1, sul2, ermB, tetA, tetM, tetO, tetQ, tetS, tetW, tetX, cfr, cmlA, fexA, fexB, floR, oqxB, qepA, qnrA, qnrB, qnrD, qnrS.

7 Genes detected: mecA, mecC, blaZ, tet(K), tet(M), tet(L), tet(O), aac(6)-aph(2), fusB, fusC, erm(A), erm(B), erm(C), erm(F), erm(T), msr(A), msr(B), mph(C), ant(4)-Ia, lnu(A), lnu(B), cfr, ant(6)-Ia, ant(3) (9), str, dfr(A), dfr(D), dfr(K), dfr(G), mupA, mupB, catpc194, catpc221, catpc223.

8 Genes detected: ermB, msrA, msrC, ermQ, ermT, mefA, tetM, tetL, tetO, tetS, tetA, tetB, blaCMY-2, blaCTX-M, blaDHA, blaOXA-1.

9 Genes detected st,lt(ETEC);bfp,eae(EPEC);aat, aai(EAEC), (stx1andstx2), (ipaH), (ial), kpsMII (group II capsule), papA(pilus-associatedproteinA), sfaS (S-fmbrial adhesine), focG(F1Cfmbriaeprotein); hlyD(haemolysinD), afa(afmbrialadhesine),iutA.

10 Genes detected aph(6)-Id,aph(3′')-Ib,aadA1,aadA5,aac(3)-IId),beta-lactams(blaCTX-M14,blaEC-5/8/13/15/18,blaTEM-1),erythromycin(mph(A)),quaternary ammonium compounds(qacEdelta1), sulphonamides(sul1/2), tetracycline(tet(A), tet(B)), ortrimethoprim (dfrA1,dfrA5,dfrA14,dfrA17), arsB-mob.

11 Genes detected ermB,ermF(macrolide),tetA,tetM,tetO,tetW(tetracycline),sul1,sul2(sulfonamide),aadA2(aminoglycoside),vgaA,andvgaB(pleuromutilin).

12 mcr-1 mcr-9, blaIMP, blaKPC, blaNDM, blaOXA-48-like, qnrA,qnrB,qnrC,qnrS,aac(6)-Ib, qepA.

13 Genes detected: vgA,msrC,VatD,vgbB,vgbA,ermB,vatE],vancomycin[vanA, vanB,vanC1, vanC2, vanD,vanG,vanM,vanN], linezolid[cfr, cfrB, cfrD, optrA,poxtA], nitrofurantoin[nfsA,nfsB],tetracyclines[tetA,tetB,tetC,tetL,tetK,tetM],macrolides[ermA, ermB,ermC,ermTR,mefA/E],quinolone, lukF/lukS-PV, hla,hlb and hld, eta, etb, etd2, tst.

14 Genes detected qnrA,qnrS,andaac(69)-lb-cr) andcarbapenemase(blaNDM,blaSPM,blaIMP,blaOXA-48,blaKPC, blaVIM.

15 aac(6′)-Ii, aac(6′)-Ie-aph(6“)-Ia, aph(6″)-Ib, aph(6“)-Ic, aph(6″)-Id, aph(3′)-IIIa, ant(3″)-Ia, ant(4′)-Ia, ant(6)-Ia, ant(9)-Ia, qnrE. faecalis, erm(A), erm(B), erm(C), lnu(A), lnu(B), vat(A), vat(B), vat(C), vat(D), vat(E), tet(K), tet(L),

**Abbreviations:** *E. coli, Escherichia coli*; qPCR, quantitative polymerase chain reaction; CPE, carbapenemase-producing Enterobacteriaceae; WWTP, wastewater treatment plant; ARGs, antimicrobial resistance genes; AMR, antimicrobial resistance; MDR, multidrug-resistant; ARBs, antimicrobial-resistant bacteria; GIS, geographic information system; *K. pneumoniae, Klebsiella pneumonia*; Spp., species; DWDS, drinking water distribution system; ESBL, extended-spectrum beta-lactamase; *V. cholerae, Vibrio cholera; V. parahaemolyticus, Vibrio parahaemolyticus*; US, United States; FDA, food and drug administration; *S. aureus, Staphylococcus aureus; P. aeruginosa, Pseudomonas aeruginosa; C. difficile, Clostridioides difficile*; NTCKPC, New Delhi metallo-beta-lactamase carbapenemase; RamanDIP, Raman direct inoculation procedure; RACE, rapid antimicrobial susceptibility testing by capillary electrophoresis; NOVC, Non-O1, Non-O139 *Vibrio cholerae* strains; and NTS, potential sources of nontyphoidal Salmonella.

## Culture-dependent methods

Culture-dependent methods are fundamental in the detection of AMB’s in water samples. This approach encompasses a range of techniques that allow researchers to identify resistant strains based on their growth patterns and phenotypic characteristics.

### Use of selective media for the detection of AMR

Selective media are designed to isolate specific microorganisms, including those that may harbour ARGs, by inhibiting the growth of non-target bacteria (Bonnet et al. 2020). Based on the reviewed studies, typically two main approaches are employed by studies assessing water samples for AMR: (i) the use of media intended for initial general bacterial differentiation and (ii) the use of media specifically formulated to target resistant organisms (Shaw et al. 2014, Enayati et al. 2015, Lyimo et al. 2016, Kimera et al. 2021). The use of resistance specific selective media is particularly effective for the preliminary screening of ARB, as it allows for the direct and specific detection of resistant strains. Examples of such media include CHROMagar™ ESBL and HiCrome™ ESBL agar, used to isolate extended spectrum beta-lactamase (ESBL) producing bacteria (Angeles et al. 2020, Singh et al. 2020, Zagui et al. 2023), and CHROMagar™ KPC for the recovery of CRE (Haberecht et al. 2019, Mahmud et al. 2020, Zagui et al. 2023). Additionally, general selective media, e.g. McConkey, TBX, and mTEC ChromoSelect, are often supplemented with specific antimicrobial agents for resistance screening, such as meropenem for carbapenem-resistant and cefotaxime for cephalosporin-resistant organisms (Blaak et al. 2015, Moretto et al. 2021, Asaduzzaman et al. 2022). Both approaches are typically followed by further phenotypic (e.g. Antimicrobial Susceptibility Testing) and/or genotypic (e.g. PCR, WGS) testing to identify resistant strains in cases where general selective media is used and to conduct more in-depth characterization where isolates have been recovered using resistance specific selective media.

### Antimicrobial susceptibility testing

Assessing the susceptibility of bacteria to various antimicrobial agents provides insights into the prevalence and dynamics of AMR in aquatic environments. The disk diffusion method, also known as the Kirby–Bauer method, is a widely adopted procedure which involves placing antimicrobial-infused disks on agar plates inoculated with bacteria and subsequently measuring inhibition zones to determine whether bacteria are susceptible, intermediate or resistant (Reller et al. 2009). This method is the most commonly used method in the reviewed studies, most likely due to its simplicity, cost-effectiveness and suitability for routine testing (Reller et al. 2009, Gajic et al. 2022). In addition, the disk diffusion technique is often used to simultaneously screen for specific resistance mechanisms, such as ESBL production, using the Double Disk Synergy Test and the Double Disk Diffusion Test (Shahin et al. 2019, Ahsan et al. 2022, Jeamsripong et al. 2022).

In addition to the disc diffusion method, other commonly used antimicrobial susceptibility testing (AST) methods in studies assessing AMR in water samples include the quantitative broth microdilution and agar dilution methods, both of which involve determining the minimum inhibitory concentration (MIC) of antimicrobial agents. The broth microdilution method, as used by Li et al., and Singh et al., involves observing bacterial growth in microtiter plates with serial dilutions of the antimicrobial agents to determine MIC (Li et al. 2020, Singh et al. 2020). This method is often combined with the disk diffusion method, as in studies by Li et al. and Tabut et al. as for some antimicrobial agents, such as polymyxin E and daptomycin, it provides more reliable and accurate results (Li et al. 2022, Tabut et al. 2022). The agar dilution method, used in studies by Jeamsripong et al., and Suzuki and Ushijima, involves incorporating varying concentrations of antimicrobial agents into agar plates, where bacterial growth is then assessed to determine the MIC (Suzuki and Ushijima 2016, Jeamsripong et al. 2022). While both are well standardized methods and pivotal for determining the MIC of antimicrobial agents, similar to the disk diffusion method, they are both time and labour intensive (Reller et al. 2009, Gajic et al. 2022).

The antimicrobial gradient test (E-test^®^) combines the concept of disk diffusion and MIC determination by using a gradient of antimicrobial concentrations on a plastic strip to provide precise MIC values (Reller et al. 2009). This method has been employed in studies to screen bacterial isolates recovered from water samples for susceptibility to various antimicrobial agents such as cephalosporin, lincomycin, fluoroquinolone, carbapenem, macrolide, and sulfonamide antimicrobials (Blaak et al. 2015, Shahin et al. 2019, Williamson et al. 2022). While this is a simple and accurate method to determine MICs, it is relatively more expensive than the traditional disk diffusion method and therefore generally only suited for testing a small number of antimicrobials (Balouiri et al. 2016, Gajic et al. 2022).

In recent years, automated AST systems, such as the VITEK2^®^ (bioMérieux) and BD Phoenix Automated Microbiology System (BD Diagnostic Systems) have been used in studies investigating AMR in water samples (Luczkiewicz et al. 2015, Cohen et al. 2020, Mahmud et al. 2020, Tsvetanova et al. 2022). While these systems offer rapid and standardized MIC values and are valued for their efficiency and ability to significantly reduce labour involved, they can be more costly and complex to maintain (Reller et al. 2009, Gajic et al. 2022).

Overall, traditional culture-based techniques continue to play a key role in detecting AMR in water samples as, despite their limitations, they are well established, standardized methods that enable consistency and reliability in results (Table 2). Ongoing advances, such as the automation of AST techniques and the integration of culture-based methods with molecular approaches, aim to reduce labour and time, enhance reproducibility, and enable further characterization of resistance organisms in water samples. An overview of common molecular techniques used for AMR detection in water is provided.

**Table 2.** Summary of strengths and weaknesses of current detection methods for AMR monitoring in water environments. Identified in this study through literature review.

Methods	Strengths	Weaknesses
Culture-dependent	– Ability to confirm phenotypic resistance using qualitative and quantitative methods – Simple to perform, implement and interpret – Low cost and minimal technical requirements – Well established and standardized for consistent reliability – Offers flexibility in antimicrobial selection	– Limited to viable, culturable bacteria and may not detect low levels of resistance – Labour intensive and time-consuming – Risk of false positives/negatives due to varying culture conditions and media composition – Provide limited insights into the genetic mechanisms of AMR – Typically targets specific bacteria, potentially missing others – Some bacteria have specific growth requirements that can be difficult to replicate
PCR	– High sensitivity for detecting low concentrations of ARGs	– Susceptibility to inhibitors in environmental samples can affect assay reliability
	– Specificity—precise detection of target ARGs	– Requires prior knowledge of target genes for primer design
	– Quantitative capabilities (qPCR) for measuring gene abundance	– Cannot differentiate between viable and non-viable bacteria
	– Rapid turnaround time	– Presence of ARGs does not necessarily indicate active resistance or infection
	– Applicable to various water matrices	– Limited in detecting novel or unknown ARGs without prior sequence information
	– Useful for surveillance, source tracking and impact assessment	– Complexity in data interpretation
Metagenomics	– Captures a wide range of ARGs without prior knowledge of targets	– High computational demands
	– Analyses the entire microbial community, including non-culturable organisms	– Potential biases in DNA extraction and sequencing
	– Processes large numbers of samples simultaneously	– Expensive
	– Provides relative abundance of ARGs in samples	– Quantification can be influenced by variations in DNA extraction efficiency
	– Tracks changes in ARG prevalence over time and under different conditions	– Difficulty in linking ARGs to specific bacterial hosts
	– Detects plasmids and other mobile genetic elements	– Complexity in distinguishing between chromosomal and plasmid-borne genes
	– Eliminates bias introduced by culture-based methods	– May miss low-abundance ARGs
	– Links ARGs to microbial function and ecology	– Requires integration with other omics data for functional insights

## Polymerase chain reaction

PCR has emerged as a cornerstone technique in molecular biology, particularly for the detection of ARGs in various environments, including water (Franklin et al. 2021). Given the escalating concerns over AMR and its implications for public health, PCR offers a robust method for monitoring and understanding the distribution and prevalence of ARGs in aquatic ecosystems (Yamin et al. 2023). The detection and monitoring of ARGs in water environments is critical to understand the spread and impact of resistance on public health. Water bodies, including surface water, groundwater and wastewater, serve as reservoirs and conduits for ARGs, such as *bla, mecA, vanA, sul1, tetA*, and *qnr*, originating from various sources, e.g. agricultural runoff, industrial discharge and municipal wastewater (Blaak et al. 2015, Akiba et al. 2016, Kaiser et al. 2023, Ripanda et al. 2023). The presence of these genes in aquatic environments facilitates their horizontal transfer among microbial communities, potentially leading to the emergence of MDR pathogens. PCR amplifies specific DNA sequences through a series of thermal cycles: denaturation, annealing and extension. This process is facilitated by DNA polymerase and specific primers that bind to the target ARG sequences, resulting in exponential amplification (Garibyan and Avashia 2013). The techniques sensitivity and specificity make it suitable for detecting low-abundance ARGs, such as *bla, mecA* and *vanA* in complex environmental samples (Bessa et al. 2014, Bergeron et al. 2015, Goh et al. 2022). A study by Cho et al., used PCR to investigate AMR mechanisms and plasmid diversity in MDR enterococci isolated from surface water. Screening of 51 isolates revealed the presence of 27 ARGs, including those conferring resistance to ciprofloxacin, erythromycin, tylosin, kanamycin, streptomycin, lincomycin, Quinupristin/Dalfopristin, and tetracycline. Identified resistance genes such as *erm(B), erm(C), aph(3′)-IIIa, ant(6)-Ia, lnu(B), vat(E), qnrE. faecalis, and tet(K), tet(L), tet(M), tet(O)*, and *tet(S)* indicated a diverse range of AMR mechanisms. A plasmid classification system identified 19 replicon families, with 12 different rep-families prevalent among the isolates. While ARGs common in human and animal sources were detected, many isolates lacked identifiable resistance genes, underscoring the diverse and poorly understood AMR mechanisms in environmental enterococci (Cho et al. 2020a).

In this review, PCR was extensively used to detect and quantify ARGs in various water matrices, including surface water, groundwater, wastewater, and drinking water due to its high sensitivity, specificity, quantitative capabilities, and rapid results (Table 2). PCR plays a pivotal role in several key applications. Firstly, it facilitates the surveillance of ARGs such as *sul1, tetA*, and *qnr* in aquatic environments, providing critical data for monitoring the prevalence and potential impacts of resistance (Kimera et al. 2021, Williamson et al. 2022, Bhandari et al. 2023). Secondly, PCR enables source tracking by comparing ARG profiles (e.g. *bla*TEM, *ermB, intI1*) among different water samples, thereby identifying specific contamination sources such as agricultural runoff, industrial discharge and wastewater effluents (Bergeron et al. 2015, Akiba et al. 2016, Su et al. 2018, Blaak et al. 2021, Nolan et al. 2023b). Finally, PCR assays are instrumental in assessing the effectiveness of interventions such as advanced wastewater treatment processes in mitigating the prevalence of ARGs such as *blaOXA, mcr-1*, and *aadA* in water bodies (Castrignanò et al. 2020, Mukherjee et al. 2021, Li et al. 2022).

## Sequencing based methods

Sequencing has emerged as a powerful tool for the detection and characterization of ARGs in water environment. This approach enables the comprehensive analysis of microbial communities and their resistomes without the need for culturing, providing insights into the diversity and abundance of ARGs present in water bodies (de Abreu et al. 2020). In this review, numerous high-throughput sequencing technologies, such as Illumina, PacBio, and Oxford Nanopore were employed to generate large volumes of sequence data (Cohen et al. 2020, Gu et al. 2023, Liu et al. 2023, Yang et al. 2023). Shotgun metagenomic sequencing was commonly used to capture the entire genetic content of the microbial communities (Spänig et al. 2021, Gabashvili et al. 2022, Bell et al. 2023). Common ARGs frequently identified in water metagenomic studies were genes often associated with resistance to commonly used antimicrobials such as *bla*_TEM_, *bla*_CTX-M_, *bla*_SHV_ (genes conferring resistance to β-lactam antimicrobials), *tetA, tetM, tetW* (genes providing resistance to tetracyclines), *sul1, sul2, sul3* (genes linked to sulfonamide resistance), and *aadA, strA/strB* (genes associated with aminoglycoside resistance), posing a significant public health risk (Czekalski et al. 2015, Akiba et al. 2016, Wang et al. 2020).

Several recent studies have highlighted the use of metagenomics in detecting AMR in water. For instance, a study by Su et al., examined ARGs and bacterial communities in source water, drinking water treatment plants and tap water in China. In source water, *floR* and *sul1* were the most prevalent among 27 targeted ARGs. Correlation analysis identified *sul1, sul2, floR*, and *cmlA* as potential indicators for ARG presence. Tap water had a much lower abundance of ARGs compared to source water. Sand filtration and sedimentation effectively reduced ARGs, but granular activated carbon filtration increased their abundance. The study also suggested that *Pseudomonas* may play a role in ARG proliferation in treatment plants. Although significantly reduced, bacteria and ARGs remained in treated tap water, indicating a need for optimized treatment processes (Su et al. 2018). Another study by Akiba et al., investigated the distribution and relationships of AMR determinants among extended-spectrum-cephalosporin (ESC)-resistant and carbapenem-resistant *E. coli* isolates from aquatic environments in India, water samples were collected from rivers and sewage treatment plants across five Indian states. A total of 446 *E. coli* isolates were randomly obtained, with 169 (37.9%) demonstrating resistance to ESC and/or carbapenem. From selected isolates, 49 plasmids were extracted and their whole-genome sequences analysed. This analysis revealed 50 resistance genes and identified 11 different combinations of replicon types among the plasmids. Network analysis indicated that plasmids sharing replicon types tended to form communities based on gene similarity, resulting in four distinct communities containing between 4 and 17 plasmids each. Notably, 3 of these communities included plasmids from different Indian states, indicating that interstate dissemination of ancestral plasmids had occurred (Akiba et al. 2016).

Although metagenomics has numerous strengths and has proven to be a valuable method for detecting AMR in water (Table 2), several challenges remain. These include the complexity of data analysis, the need for standardized protocols and the interpretation of the clinical relevance of detected ARGs. Moreover, linking ARGs to specific bacterial hosts remains a significant challenge, although advancements in single-cell genomics and metagenome-assembled genomes are promising (Liu et al. 2022). Future research should focus on longitudinal studies to track changes in the resistome over time, the impact of interventions such as advanced wastewater treatment processes and the development of global surveillance networks to monitor AMR spread through water systems. Integrating metagenomics with other omics approaches, such as transcriptomics and proteomics, could also provide deeper insights into the functional dynamics of ARGs in aquatic environments.

## Challenges and future perspectives in ARG detection in water

Aquatic environments can often be overlooked as a reservoir of AMR but recent publications showing its significance demonstrate the need to be able to appropriately monitor water sources for this potential risk (Morris et al. 2024). There are, however, numerous challenges associated with the detection of AMR targets in different water sources. It is critical that these challenges are addressed and investigated due to the significant threat which AMR represents by making the treatment of infections more difficult and significantly increasing the risk of the spread of disease causing illness and even death (WHO 2015). The challenges associated with determining whether or not a water source is a reservoir for AMR are multifaceted and include technological, methodological, and ecological. In this section, we will delve into these aspects further.

One of the main issues with detection of AMR targets in waters is that of collecting actual water samples and ensuring that the sample collected is representative of what is by nature a heterogeneous environment. Sample volume for example is something which can cause logistical problems for AMR target detection. Generally, very large volumes are required to ensure that bacteria harbouring ARGs which can be present at low levels are captured. Some methods use up to 30 l (Morris et al. 2016) which can mean multiple collection containers, pooling of the samples and followed by filtration to capture targets of interest from the sample. In some cases, enrichment of these filter captured samples is carried out which is beneficial in terms of increasing the bacterial load and hence the organisms carrying AMR targets but it can also lead to bias potentially causing unnecessary concern. Attempts are being made to reduce the sample size by the introduction of new bacterial capture methods by which demonstrated the potential of using 100 ml water samples for detection of Shiga toxin-producing *Escherichia coli* (STEC) in water sources (Alfahl et al. 2024). The same methodology could potentially be applied to the detection of bacteria carrying ARGs. This would negate the need for collection of very large samples and reduce the potential for bias in analysis. In addition to a reduction in the volume using such a method capture of the organisms of interest onto a filter can also reduce the problem of inhibitors in the sample. Water often contains inhibitors of molecular detection methods. These include things such as heavy metals, humic acids, and organic matter. Filtration offers potential to reduce if not eliminate these inhibitory substances without losing the target DNA from the sample. Capture and lysis methodology could be tagged with molecular detection methods or next generation sequencing depending on the required output.

Temporal and spatial variability is another challenge when determining presence of AMR targets in water. Concentrations of targets in aquatic environments can vary significantly depending on location and time, and even after significant weather events such as storms. Storm water runoff, e.g. can contain high levels of pathogenic and resistant organisms from land. Sampling at, after, or around such events can lead to an over estimation of the bacterial and AMR load. Therefore, multiple sampling events must be undertaken to ensure there is an accurate representation of ARGs in the water source.

A number of methods and technologies have been utilized to detect ARGs in aquatic environments. As already mentioned in this review molecular detection methods are among the most adaptable for monitoring applications. Whole genome and metagenomic analysis have also been utilized but have a longer turnaround time than molecular amplification methods. These technologies all have advantages and disadvantages which are worth considering depending on the application (Table 2). Real-time PCR and quantitative real-time PCR both offer the potential to detect targets at low copy numbers and have a reasonably rapid turnaround time, but the technology can be prone to impact from inhibitors and can only detect the specific targets being amplified rather than provide an overview of what microbial communities and ARGs that might be present in any given sample. q-PCR also has the advantage of being able to give an output for the quantity of a given target in the sample assuming that the assay has been developed is efficient. Loop-mediated isothermal amplification (LAMP) is another alternative technology which offers some advantages over real-time PCR. It is extremely rapid and capable of generating results within 15 min. It is isothermal which negates the need for expensive thermocycling equipment and is more tolerant to inhibitors than PCR (Nwe et al. 2024).

Whole genome sequencing can provide a massive amount of information with regard to the microbial communities and AMR target diversity within a sample. However, there are a number of aspects which mean it is more labour intensive than molecular detection technologies. These include the extraction of high quality DNA which must be quantified to ensure there is sufficient material present to proceed. In many instances, laboratories tend to outsource their sequencing activities making for a longer turnaround time and once reads are generated, there is a requirement for bioinformatics analysis. The entire process can be expensive in comparison to direct detection technologies.

To date, there has been a lack of standardization of protocols for the detection of AMR targets in water. Every aspect from collection, DNA extraction, amplification, and detection needs to be standardized and in order for results to be reliable and comparable. There have been a number of publications on the standardization of methods for detection of AMR targets. As far back as 2015, the WHO published the WHO Global Action Plan for AMR in which a guide for AMR surveillance was set out. The report recommended a culture based method for detection of ESBL-producing *E. coli* as a potential candidate for AMR monitoring (WHO 2015). However, the use of a single organism limits the investigation and does not represent the full microbial community which may be present in a sample. Additionally, non-culturable organisms carrying resistance genes can be problematic and can contribute to AMR in a water source which cannot be detected except using sequencing methodologies.

More recently, Franklin et al. compared culture and molecular methods for the detection of AMR in surface waters. The authors concluded that no single method alone was adequate for determination of AMR and that a combination of methods, including culture remained necessary, in order to generate optimal information. They also set out the lack of standardization across laboratories for the various methods (Franklin et al. 2021). Furthermore, Keenum et al. have proposed a framework which they say assists with aligning target and method selection with the objectives of the monitoring programme and lays out a pathway for standardizing approaches for AMR monitoring. The authors have yet to demonstrate practical application of their proposal (Keenum et al. 2022). Other publications have proposed an adaptable global framework for routine monitoring of AMR in water and wastewater with the suggestion that there is a need for a short, medium, and long term approach to implementing standardized protocols. The authors also spell out the need for international policies and agreements in order to progress implementation of monitoring programmes (Cutrupi et al. 2024).

Clearly the detection of ARGs in aquatic environments is of critical importance but the range of water sources including rivers, wastewater, seawater, drinking water and lakes, in which these targets can be found is broad and can pose challenges. A tailored approach is therefore required to enable monitoring. One of the main issues with monitoring of water sources for AMR is the presence of complex mixed microbial communities and the difficulty in implementing methods which identify exactly which communities are contributing to resistance. Additionally, horizontal gene transfer between bacteria complicates the situation further. The aquatic environment can therefore be in constant flux making AMR detection difficult to interpret regardless of the method used. The presence of AMR targets in water does not necessarily equate to AMR in pathogens and so data interpretation needs to be carefully assessed. Added to this is a further layer of complexity caused by the composition of various water sources. Lakes, wastewater, drinking water, etc. all have a unique composition and so specifically tailored approaches for detection are required. This is particularly the case of detection in effluent and seawater for instance.

A number of studies have aimed to determine the link between AMR in water and carriage and infection in humans. For example, Leonard et al. surveyed bathing waters for AMR and aimed to determine the association between human exposure and colonization. The authors found that water users were at risk of exposure to colonization by resistant *E. coli* they were exposed to showing the role of the environment in transmission (Leonard et al. 2018). However, a further study was carried out by Farrell et al. to determine the prevalence of ESBL-producing Enterobacterales (ESBL-PE) and CRE in recreational water users and matched controls, demonstrated the occurrence of ESBL-resistant organisms in healthy participants in Ireland but showed a decreased prevalence of colonization (Farrell et al. 2023). These studies outline the challenges associated with detecting AMR in water sources and the difficulty in interpreting the potential risk to human health which may result.

There are a number of other challenges associated with detection of AMR targets in water source. These include appropriate assessment of the risk which is posed by gene targets associated with AMR and any potential impact on human health. Such an assessment can only be done using a multidisciplinary approach combining microbiology and molecular biology, epidemiology, and the environment sciences. An awareness among all stakeholders, including policymakers, industry, and the public around AMR in water and the potential risks associated with it will be critical to gaining support for any regulatory measures and further research.

While there exist many challenges in detecting of AMR in water, advancements are being made which will progress the field. There are new technologies which are enabling easier detection in water, including portable sequencing technologies such as those available from Oxford Nanopore. The technology has been trialled in various high complexity environments and the company also offer bioinformatics solutions. Portable LAMP systems also offer the potential to detect AMR threats onsite, enabling more rapid decision-making (Hassan et al. 2023, Higgins et al. 2023). In addition to this, open global collaboration and data sharing are key to interpreting patterns of AMR dissemination and interpreting the AMR landscape and dissemination (Hendriksen et al. 2019, Munk et al. 2022). Platforms and databases which provide open access can enhance the sharing of methodologies, data and findings. Finally, integrating water quality and AMR monitoring systems could provide data which would inform the development of mitigation strategies.

In conclusion, the detection of AMR in water poses many challenges which range from technological to regulatory domains. A multidisciplinary approach and global effort from scientists, policymakers, and stakeholders are required to address these challenges. The aim at all times must be to mitigate the spread of AMR in aquatic environments in order to reduce the risk to human and animal health.

## Supplementary Material

qvae034_Supplemental_File

## Data Availability

The data underlying this article are available in the article and in its online supplementary material.
