# Supplementary material for: Antimicrobial resistance detection methods in water environments: a scoping review
Source: Sustain Microbiol. 2024 Nov 28;1(1):qvae034. doi: 10.1093/sumbio/qvae034 (PMC13375382; doi:10.1093/sumbio/qvae034)
Supplement: Supplementary file 1 [file qvae034_Supplemental_File.docx]

**Antimicrobial Resistance Detection Methods in Water Environments: A Scoping Review (Supplementary data)**

Zina Alfahl^1,2^*, Alexandra Chueiri^2,3^, Shaunagh Carolan^2,3^, Gabriel Darcy^2,3^, Nadia Hussain^4,5^, Niamh Cahill^1,2^, Louise O’Connor^2,3^

^1^ Antimicrobial Resistance and Microbial Ecology Group, School of Medicine, University of Galway, Galway, Ireland

^2^ Centre for One Health, Ryan Institute, University of Galway, Galway, Ireland

^3^ Molecular Diagnostics Research Group, College of Science & Engineering, University of Galway, Ireland

^4^ Department of Pharmaceutical Sciences, College of Pharmacy, Al Ain University, UAE

^5^ AAU Health and Biomedical Research center, Al Ain University, Abu Dhabi, UAE

*Corresponding author at:

Antimicrobial Resistance and Microbial Ecology Group, School of Medicine, University of Galway. Email address: [zina.alfahl@universityofgalway.ie](mailto:zina.alfahl@universityofgalway.ie)

**A1. Search terms**

**Table S1** Search terms and number of new studies retrieved from each database

| **Keywords** | **Pubmed** | **Science Direct** | **Web of Science** | **Google Scholar** |
| --- | --- | --- | --- | --- |
| AMR AND water | 55 | 2 | 20 | 7 |
| Antimicrobial resistance genes AND water | 9 | 9 | 16 | 1 |
| Culture AND AMR OR Antimicrobial resistance AND water | 0 | 7 | 3 | 1 |
| Molecular AND AMR AND water | 0 | 7 | 0 | 2 |
| **Total** | 64 | 25 | 39 | 11 |
| **Total after duplicates removed** | 64 | 19 | 27 | 5 |
